# Effectiveness of acupuncture therapy for preventing emergence agitation in children: A protocol for systematic review and meta-analysis with trial sequential analysis

**DOI:** 10.1371/journal.pone.0264197

**Published:** 2022-03-29

**Authors:** Daisuke Nakajima, Takahiro Mihara, Toshiyuki Hijikata, Makoto Tomita, Takahisa Goto

**Affiliations:** 1 Department of Health Data Science, Yokohama City University Graduate School of Data Science, Yokohama, Japan; 2 Department of Anesthesiology, Yokohama City University, School of Medicine, Yokohama, Japan; Ospedale Sant’Antonio, ITALY

## Abstract

Pain, autonomic distress, and emergence agitation occur commonly in children undergoing general anesthesia. While acupuncture therapy has been reported to effectively reduce such pain and autonomic distress in children, its effect in preventing emergence agitation remains unclear. Therefore, we will conduct a systematic review and meta-analysis with trial sequential analysis to evaluate the effect of acupuncture therapy in preventing emergence agitation in children undergoing general anesthesia. **Methods and analysis** This protocol was prepared according to the 2015 Preferred Reporting Items for Systematic Reviews and Meta-Analyses (PRISMA) for Protocols guidelines. We will conduct a search for randomized controlled trials that evaluated the effect of acupuncture therapy in preventing emergence agitation. The following databases will be searched for relevant articles: MEDLINE, CENTRAL, Embase, and Web of Science; four pre-registration sites will be accessed from inception to April 1, 2021. No language restrictions will be applied. Two authors will independently scan and select eligible studies, extract the data, and assess the risk of bias. The incidence of emergence agitation will be combined as a risk ratio with a 95% confidence interval using a random-effect model. The I^2^ statistics will be used to assess heterogeneity. We will evaluate the quality of the clinical trials using the Cochrane methodology and assess the quality of evidence using the Grading of Recommendation Assessment, Development, and Evaluation approach. If appropriate, a trial sequential analysis will be performed. **Expected outcomes** This meta-analysis will be the first to evaluate the effect of acupuncture therapy in preventing emergence agitation in children. The findings from this meta-analysis have the potential to reveal pivotal factors that affect the clinical effect of acupuncture therapy, thereby supporting the optimization of acupuncture therapy for emergence agitation.

**Protocol registration** University Hospital Medical Information Network Clinical Trials Registry (UMIN000040775).

## Introduction

### Description of the conditions

Emergence agitation (EA) is a significant complication in children undergoing general anesthesia, with a reported frequency ranging from 10% to 80% [1]. Sikich and Lerman [2] defined emergence delirium, which is a significant cause of EA, as follows: “a disturbance in a child’s awareness of and attention to his or her environment with disorientation and perceptual alterations including hypersensitivity to stimuli and hyperactive motor behavior in the immediate post-anesthesia period.” Restless recovery from anesthesia may increase the risk of self-injury and result in the additional adjunctive administration of sedatives and/or analgesic medications, which may delay the patient’s discharge from the hospital. Moreover, EA often requires constant nursing supervision, which strains nursing staff resources and induces stress in caregivers and families [1,3].

### Description of the intervention

Pharmacological agents, including propofol, fentanyl, dexmedetomidine, and clonidine are the conventional anesthetics used for preventing EA [4–8]. However, these pharmacological agents may often increase the adverse events such as delayed recovery, vomiting, and respiratory depression [8,9]. Therefore, alternative methods for preventing EA are desired.

Acupuncture therapy is considered an effective solution without pharmacological therapy and is reported to prevent EA without causing adverse events [10,11]; however, its efficacy remains controversial [12]. Acupuncture therapy is aimed at reducing symptoms and curing diseases by inserting fine needles into the skin to a certain depth at acupoints and manipulating them. It is among the most preferred complementary therapies in many countries [13]. Acupuncture therapy was initially developed as part of Chinese medicine with the aim of restoring the patient to the state of equilibrium that supposedly existed before the illness.

### Mechanism of action of the intervention

Several studies have reported that acupuncture therapy effectively prevents EA in children; however, its mechanism of action remains unclear [10,11]. Measures that prevent EA focus on the management of pain, opioid withdrawal, and its inhibitory effect on the central nervous system. In adults, several studies have suggested that acupuncture therapy activates the release of opioid peptides in the central nervous system; this has been associated with the relief of an array of pain conditions [14,15]. Acupuncture therapy increases the secretion of β-endorphin and reduces epinephrine, cortisol, and prostaglandin E2 levels, which are responsible for regulating visceral pain [16]. Several studies have reported effective stimulation points. The LI-4 (*he gu*) acupuncture point has been shown to improve gastrointestinal symptoms and reduce pain and autonomic distress in children [17,18]. The HT7 (*shen men*) acupuncture point reportedly reduces psychological stress and agitation in adults [19,20]; it also inhibits the central release of gamma-aminobutyric acid B, which regulates dopamine, resulting in a significant reduction in behavioral hyperactivity associated with morphine tolerance in animal models [21].

### Significance of this review

In children, acupuncture therapy has been a safe complementary or alternative medicine modality, with some degree of efficacy and low risk [22]. However, no meta-analyses have been conducted to understand the effect of acupuncture therapy in preventing EA in children [12]. Previous studies have varied in the method of acupuncture therapy, selection of stimulation points, and type of surgery.

The primary purpose of this meta-analysis is to assess the effect of acupuncture therapy on the incidence of EA in children undergoing general anesthesia. The effect was evaluated using a specific assessment tool compared with no treatment, placebo/sham, or standard care in children. The secondary purpose was to assess adverse events and conduct subgroup analyses when the heterogeneity of the included studies is high.

## Methods and analysis

We will conduct a systematic review with meta-analysis and trial sequential analysis (TSA). This meta-analysis will be performed according to the recommendations of the Preferred Reporting Items for Systematic Reviews and Meta-Analyses (PRISMA) statement [23] and the *Cochrane Handbook* [24]. Our study protocol and methods were pre-specified and registered on University Hospital Medical Information Network Clinical Trials (UMIN000040775).

### Eligibility criteria

#### Types of studies

We will include randomized controlled trials (RCTs) that evaluated the effect of acupuncture therapy in preventing EA compared with placebo, no medication, or standard care in children undergoing general anesthesia. We will exclude RCTs that did not evaluate the effect of acupuncture therapy and in which the incidence of EA was not evaluated using a specific assessment tool such as the Pediatric Anaesthesia Emergence Delirium scale and Aono’s scale. Eligibility will not be restricted by language, type of surgery, or anesthetic technique.

#### Types of participants

We will include studies that were conducted in children undergoing general anesthesia who were expected to be extubated in the operating room. We will exclude studies conducted in adult patients (age >18 years), those undergoing cardiac surgery with cardiopulmonary bypass, or those not expected to be extubated at the end of surgery.

### Information sources and search strategy

We will conduct a search in the following databases: MEDLINE, CENTRAL, Embase, and Web of Science. The reference lists of the retrieved articles will also be evaluated. In addition, we will conduct a search of clinicaltrials.gov, the European Union Clinical Trials Register, the WHO International Clinical Trials Registry Platform, and the UMIN Clinical Trials Registry. The search strategy combining free text and MeSH terms for PubMed is shown in Table 1.

**Table 1:** Search strategy for PubMed.

Number	Search terms
#1	("acupuncture"[Mh] OR "acupuncture"[tiab] OR "acupuncture therapy"[Mh] OR "acupoint"[tiab] OR "acupotomy"[tiab] OR "moxibustion"[Mh] OR "moxibustion"[tiab] OR "acupressure"[Mh] OR "acupressure"[tiab] OR "electrical stimulation"[tiab] OR "electroacupuncture"[Mh] OR "electroacupuncture"[tiab] OR "electroacupuncturing"[tiab] OR "transcutaneous electric nerve stimulation"[Mh] OR "shiatsu"[tiab])
#2	(“child”[Mh] OR “child”[tiab] OR “children”[tiab] OR “pediatrics”[Mh] OR “pediatrics”[tiab] OR “pediatric”[tiab] OR “paediatrics”[tiab] OR “paediatric”[tiab] OR “infant”[Mh] OR “infant”[tiab] OR “adolescent”[Mh] OR “adolescent”[tiab])
#3	(“anesthesia” [Mh] OR “anesthesia” [tiab] OR “anaesthesia” [tiab] OR “perioperative period"[Mh] OR "perioperative care"[Mh] OR “perioperative” [tiab] OR “intraoperative” [tiab] OR “preoperative” [tiab] OR “postoperative” [tiab])
#4	#2 AND #3
#5	(randomized controlled trial[pt] OR controlled clinical trial[pt] OR randomized[tiab] OR placebo[tiab] OR drug therapy[sh] OR randomly[tiab] OR trial[tiab] OR groups[tiab] NOT (animals [mh] NOT humans [mh]))
**#6**	**#1 AND #4 AND #5**

Two authors will independently scan the titles and abstracts of the reports identified by the variety of search strategies described above. We will use Mendeley to remove duplicate titles and abstracts from our database search. We will export the remaining titles and abstracts to the application Rayyan QCRI14 [25] and screen the titles and abstracts. If eligibility cannot be determined from the title or abstract, the full paper will be reviewed. Potentially relevant studies selected by at least one author will be retrieved and evaluated in full-text versions. The articles meeting the inclusion criteria will be assessed separately by two authors, and any discrepancies will be resolved through a discussion.

### Study records

#### Data collection

A standardized, pre-piloted form will be used to extract data from the included studies for assessment of study quality and evidence synthesis. The extracted information will include the following: (i) number of patients in the study, (ii) age, (iii) sex, (iv) American Society of Anesthesiologists’ physical status, (v) risk factors for EA, (vi) type of anesthesia, (vii) type of surgery, (viii) method of acupuncture therapy, (ix) duration and the onset of acupuncture therapy, (x) type of control group, (xi) number of cases of EA, (xii) absolute value of the EA score evaluated using a specific assessment tool, (xiii) absolute value of the pain score evaluated using a specific assessment tool, (xiv) time to extubation, (xv) post-anesthesia care unit (PACU) stay duration, and (xvi) adverse effects of acupuncture therapy. Two reviewing authors will extract the data independently, and discrepancies will be resolved through a discussion (with a third author where necessary). Missing data will be requested from the authors of the concerned studies.

#### Definition of acupuncture therapy

We defined acupuncture therapy as using both percutaneous needle therapy and noninvasive methods, such as pressure, electrical stimulation, heat lamps, lasers, and magnets. There are no limitations on variations in doses, intensity, administration, or personnel administering the intervention. The timing of initiation of acupuncture treatment was defined as the perioperative period, regardless of whether it was before or after general anesthesia.

### Outcomes and prioritization

#### Primary outcome

The primary outcome will be the incidence of EA evaluated using a specific assessment tool. We will follow the definitions of the incidence of EA according to the criteria established in each study. In cases where EA will be classified according to severity, we will identify and calculate the total number of patients with all degrees of severity. In cases where EA has been evaluated at several time points, we will extract the data evaluated immediately after emergence (i.e., data evaluated at the earliest time point in the PACU or recovery room) to extract the data representing acute EA.

#### Secondary outcomes

The secondary outcomes will be the absolute values of EA and the pain scores evaluated from a specific assessment tool. The incidence of adverse events such as nausea, vomiting, and delayed awakening (time to extubation and PACU stay duration) will also be analyzed.

### Assessment of the risk of bias in individual studies

We will assess the risk of bias using Cochrane’s Risk of Bias tool (RoB2.0) [26] for RCTs. The RoB 2.0 assessment for individually randomized trials has five domains and one overall risk of bias domain, as follows:

1. Bias arising from the randomization process
2. Bias due to deviations from intended interventions.
3. Bias due to missing outcome data.
4. Bias in the measurement of outcomes.
5. Bias for selection of the reported result.
6. Overall risk of bias.

The risk of bias will be assessed as “low,” “some concern,” or “high” in each domain.

### Data synthesis and statistical analyses

Statistical analyses will be performed using the R statistical software package, version 4.0.0 (R Foundation for Statistical Computing, Vienna, Austria). We will compare dichotomous outcomes between the groups using the risk ratio and continuous data using the mean difference (MD) and the corresponding standard deviation (SD). We will summarize the risk ratio and mean difference with a 95% confidence interval (CI); if the 95% CI includes a value of 0 or 1 for continuous or dichotomous data, respectively, we will consider the difference to be statistically insignificant. We will use a random-effect model (DerSimonian and Laird method [27]) to combine the results and the Hartung-Knapp-Sidik-Jonkman adjustment method [28] for the random-effect model in cases where the number of studies is small (i.e., less than 10). Heterogeneity will be quantified using the I^2^ statistic; significant heterogeneity will be considered when the I^2^ statistic exceeds 50%. We will conduct subgroup analyses to explore the possible causes in cases with high heterogeneity. The forest plot will be used to represent the effect of treatment graphically. A small study effect will be assessed using a funnel plot and Egger’s regression asymmetry test [29] and will be considered positive if p < 0.10 in the regression asymmetry test.

### Subgroup and sensitivity analyses

We plan to conduct subgroup analyses according to the following predefined factors when the I^2^ statistic exceeds 50%: (1) method of acupuncture therapy, (2) selection of points (unilateral or bilateral), and (3) type of surgery. Sensitivity analysis excluding studies with high risk of bias will be performed for primary outcome.

### Trial sequential analysis

For the primary outcome, TSA will be performed to correct for random error and repetitive testing of accumulating and sparse data [30,31]. TSA monitoring boundaries (i.e., monitoring boundaries for meta-analysis) and required information size (RIS) will be quantified, and adjusted CIs will be calculated. The risk of type 1 error will be maintained at 5% with a power of 90%. A reduction in the risk ratio by 25% will be considered as clinically meaningful. If the cumulative Z-curve does not cross the TSA monitoring boundaries, we will downgrade the quality of evidence due to inaccuracies in the results.

### Summary of evidence

We will grade the quality of evidence of the primary outcomes using the Grading of Recommendations Assessment, Development, and Evaluation approach [32,33] with GRADEpro software. Judgments of the quality of evidence will be based on the presence or absence of the following variables: limitations in study design, inconsistency, indirectness, inaccuracies in the results, and publication bias. The quality of evidence for the primary outcomes will be graded as very low, low, moderate, or high.

## Limitations and implications

This will be the first systematic review to evaluate the effect of acupuncture therapy in preventing EA in children. However, this protocol for the meta-analysis may have a few limitations. We will include studies that are related to any type of acupuncture therapy; however, if there are only a few studies on each type, we will not indicate the best acupuncture method.

The findings from this meta-analysis will indicate whether acupuncture therapy may prevent EA in children. In addition, it has the potential to reveal pivotal factors that affect the effect of acupuncture therapy in preventing EA. If the findings from this meta-analysis are inconclusive, new hypotheses can be generated around which new RCTs can be designed and conducted.

## Ethics and dissemination

This systematic review will be published in a peer-reviewed journal. Any significant changes to this protocol will be noted with a description of the change, the corresponding rationale, and the date of the amendment. The results will also be presented at relevant conferences. The data used do not include individual patient data; thus, there are no patient privacy concerns. There are no restrictions on publicly sharing data.

## Supporting information

S1 Checklist PRISMA-P (Preferred Reporting Items for Systematic review and Meta-Analysis Protocols) 2015 checklist: Recommended items to address in a systematic review protocol*. (DOC)
